# “A story with gaps”: An interpretative phenomenological analysis of ICU survivors’ experience

**DOI:** 10.1371/journal.pone.0264310

**Published:** 2022-03-03

**Authors:** Cécile Flahault, Christel Vioulac, Léonor Fasse, Sébastien Bailly, Jean-François Timsit, Maité Garrouste-Orgeas

**Affiliations:** 1 Laboratory of Psychopathology and Health Processes, Paris University, Paris, France; 2 Grenoble Alpes University, INSERM, CHU Grenoble Alpes, HP2, Grenoble, France; 3 Paris University, IAME, INSERM, Paris, France; 4 Department of Biostatistics, Outcomerea, Paris, France; 5 Medical ICU, Bichat University Hospital, Paris, France; 6 Medical Unit, French British Hospital, Levallois-Perret, France; 7 Palliative Care Unit, Fondation Diaconesses Reuilly, Rueil Malmaison, France; University of Bristol, UNITED KINGDOM

## Abstract

ICU survivors may experience various long-term sequelae, recognized as Post-Intensive Care Syndrome, that includes psychiatric symptoms: anxiety, depression, and post-traumatic stress disorders symptoms (PTSD). While it was hypothesized that an ICU diary could help patients after discharge, improving their hospitalization memories and quality of life, it is unclear whether it may reduce psychiatric disorders, in particular PTSD. We performed a qualitative exploration of survivors’ subjective experience of their ICU stay, their representations, memories, meaning-making of their experience and use of their ICU diary. Five participants (ICU survivors, 3 men and 2 women, who received a diary) were included in this study. We conducted non-directive interviews 6 months after discharge. These interviews were transcribed and analyzed using Interpretative Phenomenological Analysis. Major recurring themes of discourse included: (1) The nightmare of the ICU experience: from an impression of vagueness to dispossession, (2) The positive image of health-care workers during intensive care, (3) The place of the relatives and health-care workers’ writings in the diary: either a support or a barrier, (4) The difficult return back home, and daily life after intensive care. Participant’s representation of their ICU experience seemed to reflect the meaning they had given it through their own reflections and that of health-care workers in the diary. For some participant, the diary was associated to the pain and strangeness of the ICU experience; therefore, their recovery required them to take some distance with it. The ICU diary allowed participants to construct their illness narratives, and to become aware of the presence and support of health-care workers. The diary was also perceived as the witness of a period they wished to forget.

**Trial registration:** NCT02519725.

## Introduction

In the past several years, the survival rate of patients admitted to an intensive care unit (ICU) improved considerably [1, 2]. Consequently, the challenges of ICU survivorship have become an increasingly critical focus in research [3]. Both quantitative and qualitative studies have shown that a significant number of ICU survivors experienced a modification of their quality of life [4, 5] and developed cognitive and psychiatric impartments during and after their hospital stay such as impaired memory and executive function, depression [6, 7] and Post-Traumatic Stress Disorder (PTSD) [8–11] individualized as Post Intensive Care Syndrome (PICS), which includes physical, cognitive, and psychological impairments [12]. Comorbidities, hypoxia, hypotension, anemia, dysglycemia, sleep deprivation, medications like benzodiazepines were reported as favoring the pathogenesis of cognitive and psychological morbidity [13].

To minimize the detrimental consequences of an ICU hospitalization, several countries have used an ICU diary, provided to patients after ICU discharge, to help them recall the actual chain of events during their hospitalization and construct their illness narratives [14–16]. The diary is filled out by both ICU caregivers and family, with the objectives of reducing survivors’ memory gaps, re-establishing a sense of reality, providing an understanding of their illness severity, and setting realistic goals for a meaningful recovery [17]. Currently, the usefulness and utility of the ICU diary is a matter of debate [11, 18–20]. A meta-analysis [19] has recently confirmed that, while ICU diaries do seem to improve memories of the ICU hospitalization and reduce the risk of depression, it is not clear whether they are effective in reducing psychiatric disorders in patients after hospital discharge. Despite the valuable scientific contribution of the above-mentioned studies, very limited empirical research has investigated the subjective experience of ICU survivors [21], that is, not in terms of a presence or absence of symptoms, but rather in terms of representations, memories, and meaning-making of the experience.

To understand the issues faced by ICU survivors after they leave the hospital, we designed a qualitative study aiming to (1) capture the intimate experience of ICU patients, (2) understand how they make sense of this particular experience, and (3) explore their experience and representations of an ICU diary. Given the severity of long-term sequelae associated with ICU hospitalization [6], insights into subjective difficulties, particularly related to the adjustment and meaning-making processes, should allow a better understanding and help to target prevention interventions.

## Participants and methods

### Participants

This is a ancillary study of the multicenter randomized ICU diary study exploring the impact of an ICU diary on the psychological consequences of an ICU hospitalization in mechanically ventilated patients for more than 48 hours [18]. For this ancillary study, among the interviews of ICU survivors of the main study [18], a sample of 5 patients with the greatest variety of meaning- making process were selected to take part in a non-directive interview by phone. This variety refers to the purposive nature of the sampling. It is not linked to the heterogeneity of the sample (regarding gender, age, cause of hospitalization for instance) but rather to the way the participants gave meaning to their experience. The study (#2015-A00700-49) was approved by the ethics committee of the Necker University hospital, Paris, France (2015-05-01-SC), the CNIL (Commission Nationale de l’Informatique et des Libertés; # HZB1693034n) and by the CCTIRS (Comité Consultatif sur les Traitements de l’Information en matière de Recherche dans le domaine de la Santé; # 15.1004). Prior to the interview, participants provided their signed informed consent by mail. Oral consent was obtained also the day of the interview.

### Data collection and analysis procedures

A female psychologist (CV) dedicated to the study called the 5 participants in 2018, 6 months after ICU discharge. She has a researcher status and did not know the participants before the study. Interviews were audio-recorded and transcribed. We chose the Interpretative Phenomenological Analysis (IPA) technique to explore their content [22, 23]. This method was designed to understand the complex system of meanings attached to a unique, subjective and eminently intimate phenomenon [22–24]. According to IPA recommendations, a small sample (1–8) is required in order to highlight their intimate meaning making process related to this experience [24]. There was no pre-established script, only one question “Can you talk to me about your ICU experience?” was asked. The interview was not directed, reformulations were used to go deeper, with the constant goal to try to obtain spontaneous speech about the experience of surviving. If the participant did not spontaneously mention the diary, CV asked them how they experienced it. This method leads to a double hermeneutic: the researcher attempts to make sense of the way the subject makes sense of his or her own subjective experience [22–24]. The researcher sticks closely to the subject’s discourse and uses knowledge from the literature to propose an in-depth analysis. The IPA’s aims are not to develop general rules but rather to remain attentive to the subtleties of experience, in all its complexity. A standardized procedure ensured the IPA’s scientific rigor. CF identified discourse themes (sufficiently characterized topics, implying that the researcher could decipher a common and stable meaning in them). Then, she assessed the connections between subthemes, and identified the major themes, which are described in the results section. Lastly, she produced an interpretative account, highlighting and analyzing the experience through experiential themes and by illustrating the discourse. Then, the main themes and the interpretative account were reviewed by CV and LF (clinical psychologist and expert in IPA). Throughout this study, we pay close attention to meet the scientific rigor criteria established for qualitative analysis [25, 26]. We followed the Consolidated Criteria for Reporting Qualitative Research (COREQ) checklist [27]. To simplify the data analysis process, the data were anonymized only after data analysis was completed.

## Results

Five participants took part in this study, 3 men and 2 women, aged from 24 to 72 years old. Their characteristics are described in Table 1. The median (IQR) number of pages of their ICU diary was 13.5 (30–42.5). The participants read their ICU diary a median (IQR) of 10 (10–12.5) times.

**Table 1:** Characteristics of the participants.

Name	Age	Gender	SAPS ^a^	Length of stay in ICU (days)	Reason for ICU hospitalization	Duration of sedation (days)	Mechanical Ventilation (days)
B	72	female	47	9	Acute respiratory failure	5	8
M	65	female	38	10	Septic shock	9	10
E	36	male	34	10	Acute respiratory failure	8	10
A	24	male	60	8	Coma	4	5
P	65	male	60	13	Hemorrhagic shock	7	8

^a^ SAPS: Simplified Acute Physiologic Score indicates the severity during the first 24 hours after ICU admission.

### Analysis of major themes

The 4 major themes depicting the ICU experience of participants are presented below. These are followed by an interpretative account, which condenses the meaning-making processes of the participants and is the last step of the IPA.

#### The nightmare of the ICU experience: From an impression of vagueness to dispossession

All participants described an experience of strangeness, in particular because of the delirium or hallucinations they remembered during their stay in intensive care:

« I was out of it, I was delirious…once… I thought I was seeing my wife next to me, close, not far away. But it was not real, she was not there. » *P*, *male*, *65 years old*

Strikingly, the words “I was out of it” were mentioned by several participants depicting an experience of dissociation or dispossession.

*«* I am telling you, either I was completely out of it. I was saying nonsense, because I was seeing things. » *B*, *female*, *72 years old*
« In dreadful violence, I saw my daughter slaughtered in front of me, twice. And I, I have… the friends who came to see me, or all of the people close to me… had disasters at home, were responsible for fatal accidents or themselves seriously injured, or… really terrible things, terrible, terrible. » *M*, *female*, *65 years old*.

Some participants reported their impression that treatments had altered their emotions:

« I feel like the anesthetics had… some kind of power to… to… to make our emotions… much more intense. Because when I was euphoric, I was euphoric when there was no reason to be. But on the other hand, when I was stressed, uh… mind you, I am not stressed by nature. But when I was stressed, I was really stressed. » *E*, *male*, *36 years old*

What really dominates here is the feeling of fluctuating identity as one can experience in a nightmare or in a traumatic experience.

The situation was therefore colored with a negative tinge and lead to a dispossession from what really happened, more as memories start to fade. "Uh… well, frankly, it is starting to become vague. » *A*, *male*, *24 years old*

#### The positive image of health-care workers during intensive care

Regarding the resuscitation period, all participants underlined the important role of healthcare providers and that they had positive memories of the care they received during hospitalization. The support provided by healthcare providers is perceived as very positive in this confusing experience:

*A*, *male*, *24 years old*: « Caregivers in general. In fact, these people left a particular mark on me… and if you want, good memories… well, good memories… among things that stand out positively, it was… well, it was these people. »

The words of A. underlined the buffer effect of this professional care against his distressful experience.

*M*, *female*, *65 years old*: « Yes. As short as my experience in intensive care was, yes. They were, everyone was extremely… yes, yes. From the few memories I have, hum. Yes, yes, people were very kind, they made sure we didn’t suffer »
*B*, *female*, *72 years old*: « The staff: caregivers… were absolutely wonderful. I think I told you. Very attentive to me, of course, with all the devices I was connected to. And, it seems, very kind to my family»

It seemed that each participant remembered positive moments associated with the caregivers, both for him- or herself and for relatives. These positive reports differed from the confusion and violence of personal memories and even sometimes with the negative memories associated with some moments of care. However, the latter were balanced by a form of recognition addressed to caregivers. In this context of self-dispossession (i.e. the participants reported an impression of having lost a moment of their life… that others have experienced. There is a part of their life that escaped them), human and relational qualities displayed by professional caregivers had a high importance for participants.

#### The place of the relatives and health-care workers’ writings in the diary: Either a support or a barrier

Two participants spontaneously mentioned the ICU diary when they spoke about their memories.

*M*, *female*, *65 years old*: spontaneously described the effect that her state had produced on her caregivers and relatives, which she read in the diary:
« There is a person named Fabien who very kindly wrote in my diary, telling me that they were happy to see I was reacting at last. Uh…well, because they had to give me this treatment twice…well, I am not sure what it was [..]. The medical resident, yes, he has written it down. My kids, my husband have written a few words. But, my husband, simply was asking me to come back (laughs). ‘Come back, come back soon.’ That’s all he was writing, poor guy. Other than that, the kids were very happy. One of them said I was moving too much. But that I was not conscious of that. But this diary is very important. Well, at least, for me, as I have told you I have stayed only very shortly, uh…. nothing extraordinary happened […]. And it is very important for a patient to be able to read this diary on a day-to-day basis. Because this way we have, … well at least I do not have the impression to have lost days of my life…»

This extract also showed the reassessment of the passing of time by the participant: this period of time seemed short to her, and we observed how she trivialized it, during which, according to her « nothing extraordinary happened », even though the violence of the episode contained all the characteristics of an extraordinary moment in the sense of trauma. We could thus hypothesize that a participant attempted to appropriate this period of time through trivialization, which the diary can strengthen or contradict. In *M (female*, *65 years old)* discourse, it was the link with her relatives that the diary emphasized.

*M*, *female*, *65 years old* insisted on the importance for her to feel the close presence of her relatives while she was unconscious:
« Well, all my children were there, and, well, there is the diary. I know everyone was close to me. […]. And I know a little bit what each one has felt».

She re-read the diary with the purpose of remembering and connecting with her loved ones: « Yes, but as I told you, as I have read it several times, this or that passage, the messages of the grandchildren. I think it brought me closer to my people. Well…of my grandchildren. Closer to my husband too. This ‘come back soon’ from my husband, on the second day… it says what it says.”

*M*, *female*, *65 years old* then discussed her reading of the diary and the evolution of her perception of it, starting from a distant reading (as if it was not about her and rather "a novel" that she could read from a distance): " At the beginning, I read it a few times. A bit like a, like a novel (laughs). And then afterwards, the more I read it, the more I read it, and the more felt emotional and… (her voice sounds moved). And I realized… (pauses and cries). Because… because you know, it’s still very, very, very serious."
*M*, *female*, *65 years old* underlined the painful confrontation with the report written by the doctor and the emotional experience of her relatives, which highlights the severity of her condition:

"Well… I don’t know to what extent, but… that’s just it. To see that the doctor himself… the surgeon, the one who wrote the first message, saying that…it hit me. And then after that, the suffering of my relatives (sounds very moved). The worry. And then, I saw my daughter, I have, I have friends who came from far away. That’s it, it was… it was serious. " It was here clear what both moved and supported her in the words of her relatives and friends, but also how these words made her realize the severity of her condition.

When asked about the diary, *E*, *male*, *36 years old* emphasized its utility, but also the need for it to be kept up to date about what happened. He was indeed slightly disappointed with his reading of the diary: I don’t know, I stayed in the hospital for 15 days and I have one, two, three, I have only three full days described in it. In fact, I really have the first day. I mean… it has to be filled in… every day. And every day… let’s say, a summary of what happened during the day. Either we do it, or we don’t. Well, part of the story is missing. It’s like you start a book, you read it, the introduction, and then you finish right off with a conclusion. It’s a bit… there, that’s kind of the feeling I had. »

The other two participants did not talk about the diary without being prompted. When it was mentioned to them, they talked about it as part of what they would rather not remember about their time in intensive care:

*A*, *male*, *24 years old* seemed to know who may have written in the diary, but his preference for the moment was not to read it: "Well there are people, afterwards, who told me ‘I wrote in the little notebook, So I pretty much know who wrote. I may have surprise the day I open it, but I don’t want to. Well, I don’t want to, I don’t, it’s rehashing unpleasant things. I prefer not to, I’d rather move on, and that’s it. It’s written down, but… I do not want to think about that anymore. The day I want to, I know it’s there, but… that’s it." According to A, male, 24 years old, reading the diary would be like dwelling on a painful period of his life, which, for the moment seems to go against his wish to "move on", as if reading the diary was an obstacle to his recovery.
*P*, *male*, *65 years old* did not wish to reread it for now. To him, the diary concluded the ICU experience, and, as a record of the past, should be moved on from, to allow personal reconstruction: "I read it once, it was enough. Yes, and I don’t need to read it again. Hmmm (laughs). When did I read it? Well, I don’t know a week, two weeks, three weeks later? I do not remember. When I left, when I got home. […] No. I told myself: ‘Well, well, I haven’t gone far’, that’s all. [..] It’s okay, they did… I saw everything that was explained, yes… well, well, that’s all. No, you know I’m not a… how to say? Well I don’t like… I’m not stuck on this. Off I go, I move on, move on, move on. You see? Finally, I, how do you say? I’m not going to ponder upon this. It’s gone, hop, done. "

Although he did not wish to discuss the contents of the diary, he too emphasized the advantage of perceiving the presence of his relatives, thanks to it:

" Above all, that’s what helped me recover. Well, recover without recovering. What felt, felt good. To know that there was my wife and my son next to me. "

Thus, the diary seemed to allow participants to construct their illness narrative but also to become aware of the support of their relatives and health-care workers during hospitalization and connect with them. However, for some, the reading of the diary was a step to move beyond. For others, memories can indeed become a burden, because they may confront the participants to a temporality that they do not recognize. The reading of the diary can expose the individuals to the fact of not having been "present", during the period of ICU hospitalization in opposite of the present when they must rebuild themselves and move forward.

#### The difficult return back home, and daily life after intensive care

If the hospitalization was a period dominated by anxiety, the return to home was also described as complex:

*E*, *male*, *36 years old* pointed out the boredom and the ruminations about the ICU period and the painful memories: "And for three weeks there, I couldn’t do [sports]. So, it was already complicated to be at home, to rehash all that while being alone, to… yeah, it’s not… because I was off from work, I think two weeks, or two and a half weeks, after I got back from the hospital”
*A*, *male*, *24 years old* talked about his attempt to start an active life very quickly after discharge, because he felt better, and then about his disillusionment with fatigue persisting: "There was a time when it was much better. Well, it was… much better, I thought I was back at 100%. And when I started to have a little more intense physical activity… well I realized that in fact… well, I was still tired. In fact, I realized I did not recover 100%. I can be 100% for a few things, do things naturally and, and it’s like before. But, with the fatigue, I feel my limits come faster.”

Other participants stressed the obligation to face new limits, the feeling of being different, diminished, which can cause psychological difficulties:

*P*, *male*, *65 years old*: "I have come back to a daily life that is not completely normal, but on the whole, a normal life [..]. Well, I can’t dig in my garden like I wanted to dig my garden, I can’t jump, I can’t climb trees, you know but… no, everything’s fine. Yes, at the moment, I can no longer… I can no longer, say, garden. You know… for 6 months, I’ve been on medication, so it doesn’t… it not that it’s bothering me, but… I’m not used to medication, so… you see, that messes me up”

The difficulties in adjusting to the change and its psychological impact seemed to be linked particularly to persisting sequelae:

*B*, *female*, *72 years old*: "Well, listen, right now I’m not… I really, really, don’t often have high spirits. Not at all, really. First, I’m tired. It’s been, it’s been over 3 months now. I had a passion for gardening… because we have a garden, in the countryside, 30 km away. Well, I can’t anymore. I can’t bend anymore. It’s depressing to see how much I’m declining."

Thus, all participants underlined the difficulty of coping with memories and changes linked to their illness, as well as with the need to give up certain things when returning to their daily lives.

#### Interpretative account

This interpretative account enabled us, through the experiential themes identified in the interview, to gain an insight into the way participants made sense of their ICU experience. This account showed the convergence and discrepancies between these experiences. All interviewed participants spoke about their experience of the ICU through memories of this more or less vague and distressing period, an experience during which the support of health-care providers and that of loved ones was central. It is in this attempt of remembering their experience and finding meaning through the writings of ICU staff and relatives unveiled into the diary.

*M*, *female*, *65 years old*: "Well, let’s say, it explained, it simply explained to me… what had happened. When I had to return to the hematology department, the nurses, they told me that I hadn’t been very coherent in the past two days."

The diary shed light on blurred or distorted memories, as participants gave factual information on the hospitalization, but also as they developed their own meaning of the experience through that of others (health-care providers and relatives). Their story could have been told to the participants upon awakening, but they can also experience it live or even in real time by reading the diary.

*B*, *female*, *72 years old*: "[my daughter], who wrote ‘everyone knows about and keeps up-to-date on your situation ‘." So, as she put it, she was on vacation when my hospitalization happened, she said, ‘with Jacques, with dad and Jacques, Louis, François is flying here tonight, we are all around you’… ‘you’re not in pain, that’s the most important thing. Tonight, you’re lying on your stomach, the doctors don’t know… don’t know yet’, so, whether I’m going to make it or not."

Everyone agreed on the utility of the diary to get more information about what happened when they were not fully conscious. This implies the need for it to be fully completed:

*E*, *male*, *36 years old*: I don’t think it was duly filled in every day. I think I must have 4 or 5 messages in it. But it’s not something I’m spontaneously going to look at… to refresh my ideas or remember how it went and so on. Anyway, I think that… it’s good when you get out of the hospital to… to have a slightly external vision of how things were… how we experienced things. But frankly, I put it aside very quickly, because to read it again is also to rehash it, etcetera. So, it’s useful because… I as I was not too conscious of what was going on… it’s good, because this way we can well read what… how things happened, but with an external eye. "

As *E*, *male*, *36 years old* pointed out, it appeared that the attitude towards the diary changes after participants were discharged from the hospital and returned home. Indeed, assigning meaning to the ICU experience also involved thinking about the return to the usual daily life, of which all participants have described the difficulties. The attitude towards ICU memories therefore hold a particularly ambiguous place:

*E*, *male*, *36 years old*: "I’m telling you, a month, a month and a half, a little, a little complicated. Uh, very nervous, very sensitive, it was a little complicated for people around me because. Well, it came back all the time. As soon as I went to bed at night, as soon as… as soon as I had a moment, resting for five minutes, I had images coming up, so it was really difficult”

Also, in order to rebuild themselves after the hospitalization, the majority of participants tended to distance themselves from painful memories and therefore from the diary, sometimes up to the point of rejection:

*A*, *male*, *24 years old*: " it’s rehashing things that I don’t like. I prefer not to… I prefer to move on and that’s it. It’s recorded, but… shouldn’t be rehashed. "
*P*, *male*, *65 years old*: "So he [his grandson] read it so he could… have a written conversation. He read what was written down, and then he put in… you know, like some sort of a conscience, that is ‘I read it, well, it went well, they did that and that. The day I got home, I found it in the suitcase, I put it down…… and I put it down so well that I don’t know where I did put it. You see what I mean… "

The participants’ personal representation of the ICU experience seemed to be a composite of their own memories of the stay and of the meaning that they gave it through their own reflections and others’ account. However, the strangeness of the experience and the pain associated with the memory must be overcome to allow recovery.

## Discussion

At a time when studies on survivorship emphasize the importance of long-term psychological symptoms (depression, anxiety, PTSD) [11, 28], it seemed paramount to further investigate the psychological consequences of an acute medical episode on survivors [29]. This qualitative study of ICU survivors unveiled the importance of ICU staff and relatives during their ICU experience, their desire to forget the ICU experience and move on, the difficulties of returning to their regular life, and the different ways they took ownership of the contents the diary.

After being discharged from the ICU, our participants focused on the present and tried to distance themselves from their stay at the hospital. During the interviews, participants tended not to mention the reason for their stay in intensive care, and although they mentioned certain memories of the ICU, they seemed to experience this as a temporary episode that they were trying to forget. This need to move forward during the recovery process was reflected in the short period of time they read the diary. The diary seemed to represent a period of time, which they feel dispossessed of, but with which they did not necessarily wish to reconnect. To that extent, the ICU diary may appear as a paradoxical object. Although it was created in order to alleviate the potential traumatic impact of the absence of memories during an ICU stay, the memories presented in the diary may actually perpetuate distress [16, 20] but it could expose the participants to the traumatic effect of reading memories that were not theirs. Furthermore, as the participants mentioned, reading the diary to recollect memories seemed to go against their desire to turn towards the future. Thus, their expressed desire to move on could be explored in future studies: is this a way of appropriating the social expectations of moving forward? or to meet that of caregivers? of relatives? or a way to forget what was not consciously experienced during their sedation? The diary can appear as an object trying them to a past that they want to leave behind. The results of this study provide insight in the role of an ICU diary for the lack of improvement of physical or psychological consequences in ICU survivors during their recovery.

This desire to forget, which was expressed by a number of participants, could be related to the distress induced by the traumatic event [30], leading to avoidance, or even to memory suppression in patients with PTSD [31]. Psychological and psychiatric interventions need to take this point into account in order to minimize avoidance whilst respecting this significant anxiety. Taking care of the patients in the post ICU period lead researchers to propose programs as ICU follow-up clinics [32], follow-up visits [33], telephone-based copy skills training programs [34] or ICU diaries [19, 35]. Until now, there is no one standard model of care in place currently [36]. With regards to ICU diaries, this weak effect led us to think about how to hand over the diary to patients on the most appropriate way. There is uncertainty in existing literature as to the best time to review an ICU diary with the patient [37]. Nielsen *et al* [38] underlined that a diary written by relatives fulfilled its goal when it was shared between relatives and the patient. Patients have to be prepared to receive the diary, and sharing it should include interpreting the context and developing a reconfigured story. Thus, beyond its effects on the psychological symptoms of patients, it seems interesting to explore the relational function of diaries and their effect on the patient and his or her relatives, in order to co-create the most effective use of the diary for them. An exploration of what patients would like to do with the diary in future studies could make it possible to offer recommendations as to how to propose it: should we offer a consultation around this diary? Should the patient be encouraged to read it with his or her family? If, at first, the patient does not wish to read it, should it be provided to him or her upon request, when the patient feels the need to use it? All these questions merits to be questioned in a moment where the patient wished to forget this ICU period of time.

This study has several limitations. First, the 5 participants adjusted quite well to the ICU experience. They indeed showed some symptoms and reported distress, but they did not show psychiatric disorders such as PTSD or major depressive disorder. Despite this, this study presented a rigorous and robust qualitative methodology that highlighted in detail the subjective experience of these participants and their adaptive attempts to make sense of their experience. Second all participants were French, and our results have to be interpreted in this context. According to the French law, it is forbidden to collect ethnicity, but participants of various ethnicities may have expressed different meaning-making processes.

Third we did not collect any history of mental difficulties.

In conclusion, this qualitative study showed how participants adjusted themselves in the post-ICU period despite all issues they faced, how they wished to forget this period, and how they used the ICU diary for their recovery.
